# PLGA Multiplex Membrane Platform for Disease Modelling and Testing of Therapeutic Compounds

**DOI:** 10.3390/membranes11020112

**Published:** 2021-02-05

**Authors:** Antonella Piscioneri, Sabrina Morelli, Enrico Drioli, Loredana De Bartolo

**Affiliations:** 1 Institute on Membrane Technology (CNR-ITM), Via P. Bucci, Cubo 17/C, 87036 Rende (CS), Italy; e.drioli@itm.cnr.it (E.D.); l.debartolo@itm.cnr.it (L.D.B.); 2 Department of Environmental and Chemical Engineering, University of Calabria, via P. Bucci 45/A, 87036 Rende (CS), Italy; 3 College of Chemical Engineering, Nanjing Tech University, 5# Xinmofan Road, Nanjing 210009, China

**Keywords:** disease modelling, trans-crocetin neuroprotection, PLGA membrane, membrane systems

## Abstract

A proper validation of an engineered brain microenvironment requires a trade of between the complexity of a cellular construct within the in vitro platform and the simple implementation of the investigational tool. The present work aims to accomplish this challenging balance by setting up an innovative membrane platform that represents a good compromise between a proper mimicked brain tissue analogue combined with an easily accessible and implemented membrane system. Another key aspect of the in vitro modelling disease is the identification of a precise phenotypic onset as a definite hallmark of the pathology that needs to be recapitulated within the implemented membrane system. On the basis of these assumptions, we propose a multiplex membrane system in which the recapitulation of specific neuro-pathological onsets related to Alzheimer’s disease pathologies, namely oxidative stress and β-amyloid_1–42_ toxicity, allowed us to test the neuroprotective effects of trans-crocetin on damaged neurons. The proposed multiplex membrane platform is therefore quite a versatile tool that allows the integration of neuronal pathological events in combination with the testing of new molecules. The present paper explores the use of this alternative methodology, which, relying on membrane technology approach, allows us to study the basic physiological and pathological behaviour of differentiated neuronal cells, as well as their changing behaviour, in response to new potential therapeutic treatment.

## 1. Introduction

Advancements in tissue engineering technologies rely on the development of biomimetic models, which recapitulate the main distinctive tissue features for applications in basic research and the screening of new therapeutic treatments.

Nowadays, the global burden of neurological disease is huge, and quite often the methods of assessing cell behaviour in response to a new drug are insufficient. The key aspect for a high throughput therapeutic screening platform is the creation of a defined cellular microenvironment and specific interactions between cells, thus offering reliable tools for in vitro observations [1,2,3,4]. This challenging aspect is satisfied by the use of in vitro tools based on a membrane technology approach, which has been widely demonstrated to act as proper platform to model physiological and pathological conditions [5,6,7,8,9]. Indeed, these systems, besides portioning cells, offer them a well-controlled surrounding, taking advantage of the presence of a membrane that has been specifically tailored to better accomplish optimal tissue reconstruction [10,11,12,13]. Within the in vitro platform, polymeric membranes not only offer physical support but also drive cell adhesion and cellular contacts formation, thus acting as a biomimetic interface like an extracellular matrix. Proper validation of an engineered brain microenvironment requires a trade-off between the complexity of the cellular construct within the in vitro platform and the simple implementation of the investigational tool. The present work aims to accomplish such a challenging balance by setting up an innovative membrane platform that represents a good compromise between a proper mimicked brain tissue analogue combined with an easily accessible and implemented membrane system.

Advances in brain organoids allowed researchers to disclose important aspects of human brain development, function, evolution and disorders, making them a valid experimental tool. The complexity of neurobiology investigations requires a synergy between different approaches, including organoids, advanced microfluidics and, as in our model, investigations in material science and tissue engineering, in order to really gain new insight into neurological disease treatments [14]. Therefore, we propose a multiplex membrane system that offers precise and reliable cell responses, serving as an easy handling platform for initial and fast screening of therapeutic compounds in controlled conditions.

Another key aspect of the in vitro modelling disease is the identification of a precise phenotypic onset as a definite hallmark of the pathology that needs to be recapitulated within the implemented membrane system. On the basis of these assumptions, we studied a multiplex membrane system in which the recapitulation of specific neuro-pathological onsets related to Alzheimer’s disease pathologies, namely oxidative stress and β-amyloid_1–42_ toxicity, allowed us to test the neuroprotective effects of trans-crocetin on damaged neurons.

As mentioned before, the straightening in the use of the membrane system as a tool in neurobiotechnology resides in the presence of the polymeric membrane that recapitulates, at the cellular interface, the main cues of the native extracellular matrix. The establishment of proper cell interaction is whether it fundamentally results in proper cell adhesion, migration and movement. For the realization of the multiplex membrane tool, a Poly(D,L-lactide-co-glycolide)(PLGA) membrane was selected, whose well-defined morphological, physico-chemical and mechanical properties are adequate for supporting and boosting neuronal survival and differentiation [15]. Indeed, a previous study already showed that this membrane represents a highly cell-compatible matrix where neurons can properly organize according to a complex network with functioning cell–cell contacts and interaction. For the membrane platform setup, the PLGA membrane was located within the multiple chambers system, giving arise to the final biodevice. Since the PLGA membrane within the multiplex chamber achieves optimal neuronal differentiation, the system has the potential to serve as a highly throughput investigational platform. The well-defined condition inside the chambers, together with the cell-friendly environment, creates a physiological, relevant tool in which a wide variety of disease onset can be recapitulated in combination with the screening of therapeutic compounds. The PLGA multiplex membrane platform therefore allows for multiple investigations. On the one hand, it is possible to model different diseases, and at the same time, on the other hand, different molecules can be tested. For a straightforward validation of our system, inside it, two major hallmarks of Alzheimer’s disease neurodegeneration were mimicked, thus damaging cells through oxidative stress induction and the accumulation of beta-amyloid plaques.

Within the scenario of new molecules able to reverse neurodegeneration, emerging paths include the use of natural compounds with promising therapeutic potential. To this purpose, insulted neurons were treated with different concentrations of carotenoid trans-crocetin, which has several pharmacological properties [16,17,18,19]. Nevertheless, its neuroprotective role in counteracting neurodegeneration is poorly understood and requires further investigation. Thus, a multifunctional analysis to verify its action in reversing oxidative stress damage and the Aβ-induced toxicity was carried out.

The multiplex membrane platform allows researchers to study the integration of neuronal pathological events in combination with the testing of new molecules using a highly reproducible method. Indeed, the possibility of performing a simultaneous investigation of the different aspects involved in disease progression and treatment has a tremendous, positive impact on the value of its implementation. Control of the wide experimental settings speeds up the overall investigational time frame and maximizes the insight outcome. In the present paper, the use of this alternative methodology is described to study the basic physiological and pathological behaviour of differentiated neuronal cells and their changing behaviour in response to a potential therapeutic treatment.

## 2. Materials and Methods

### 2.1. Membrane System

**Figure 1:**
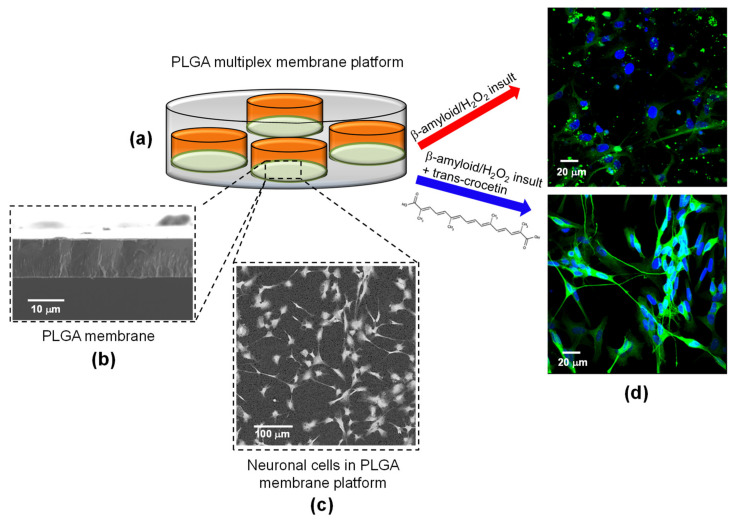
Schematic illustration of the experimental setup: (**a**) Poly(D,L-lactide-co-glycolide) (PLGA) multiplex membrane platform; (**b**) cross section of PLGA membrane; (**c**) Scanning electron microscopy (SEM) image of neurons after 7 days in PLGA membrane platform; (**d**) confocal images of neurons after toxic insult induced with amyloid-beta (Aβ) or H_2_O_2_ treatment (red arrow), and trans-crocetin administration (blue arrow). Cells were stained for neuronal marker βIII-tubulin (green) and cell nuclei marker (DAPI, blue).

The membrane system presents a kind of multiplex configuration in which cells are seeded on multiple PLGA-based membrane chambers that are located in an ordered disposition within a transparent glass device, creating a multiplex membrane platform (Figure 1). Each PLGA membrane chamber has a surface area of 1 cm^2^ and is separated from the others by a space of 2 mm, as well as having a total volume of 1 mL. The platform could contain up to 8 PLGA membrane chambers arranged in parallel.

#### 2.1.1. Preparation of PLGA Membranes

Membranes of Poly(D,L-lactide-co-glycolide) (PLGA) were prepared in a flat configuration using a phase inversion technique with solvent evaporation, as described elsewhere [15]. In brief, PLGA (10% *w*/*v*) (MW 50,000–75,000 Da, Sigma-Aldrich, St. Louis, MO, USA) was dissolved in 1.4-dioxan. The polymeric solution was cast uniformly on a glass plate by using a handle-casting knife (Elcometer, Manchester, England), gap set at 250 µm), dried at room temperature until complete solvent evaporation and then washed with distilled water.

#### 2.1.2. Characterization of PLGA Membranes

PLGA membranes were characterized in order to define their morphological, physico-chemical and mechanical properties.

The morphological properties of the membranes were characterized by scanning electron microscopy (SEM). Samples of the membrane surface and cross-section were fixed on appropriate sample holders (stub), made of conductive material and coated with graphite, and then observed using SEM (ESEM FEG QUANTA 200, FEI Company, Hillsboro, OR, USA). SEM analysis allowed for evaluation of the typical membrane structure, shape and distribution of pores over the surface.

Membrane thickness was measured with a Carl Mahr 40E digital micrometer (Esslingen, Germany). The mean diameter of membrane passing pores was determined using a Capillary Flow Porometer (CFP 1500 AEXL, Porous Materials Inc., PMI, Ithaca, NY, USA).

The physico-chemical properties of PLGA membranes were characterized in terms of the hydrophobicity/hydrophilicity of surfaces by measurement of the dynamic contact angle in advancing and receding. The instrument that was used to carry out the measurements was the Contact Angle Meter CAM 200 (KSV Instrument LTD, Helsinki, Finland), equipped with software that allows for analysis of the drop formed when the liquid is deposited on the surface of the membrane by means of an automatic micro-syringe. The contact angle measurements were carried out in different areas of the sample surface, and the final value is the mean of 30 measurements.

The mechanical properties of the membranes were measured using Zwick/Roell tensile testing machine (Ulm, Germany), via traction. The samples were cut to a size of 1 × 5 cm, fixed between two clamps in a vertical position and subjected to uniaxial tension, initially zero, and then increased to a maximum value until failure of the membrane. The instrument provides an effort–strain diagram that relates the stresses as a function of the elongations or deformations of the membrane sample. Ultimate tensile strength (UTS), Young modulus (Emod) and Elongation at break parameter (ε) were determined by analysing five replicates of the membrane sample.

### 2.2. Cell Culture

SH-SY5Y human neuroblastoma cells (ICLC-IST, Genoa, Italy) at passages P18-19 were seeded at a density of 3 × 10^3^ cell/cm^2^ in the PLGA multiplex membrane platform in a 1:1 mixture of Ham’s F12 and Minimum Essential Eagle’s medium (EMEM), supplemented with 10% (*v*/*v*) of heat-inactivated fetal calf serum, 2 mM of glutamine, and 100 μg/mL of penicillin-streptomycin, then cultured under standard conditions (37 °C, 5% CO_2_). After 24 h, the culture medium was replaced with fresh ones containing 10 µM of retinoic acid to induce the differentiation of the cell line toward a neuronal phenotype. The cultures were subsequently fed every 3 days up to 14 days.

### 2.3. Evaluation of Neuronal Differentiation

Neuronal differentiation was investigated by evaluating cell morphology by SEM and confocal analysis, and metabolic function by detection of glucose consumption, after 14 days of culture within the PLGA multiplex membrane platform.

#### 2.3.1. SEM Analysis

The cellular samples were fixed and dehydrated: after removal of the culture medium, the culture cell samples in the membrane system were initially washed with phosphate buffer (PBS) at pH 7.4, then fixed with a glutaraldehyde/formaldehyde solution (3% and 1% *v*/*v*, respectively) for 30 min. At the end of the treatment, the samples were washed again and treated with an osmium tetroxide solution (25% wt/v) for a further 30 min and finally dehydrated using ethyl alcohol solutions at progressively increasing concentrations (10–100%). The dried samples were cut, placed on special sample holders (stubs) and metallized with graphite for SEM observation.

#### 2.3.2. Immunostaining for Confocal Analysis

The differentiation of neuronal cells was analysed by Laser Scanning Confocal Microscopy (LSCM, Fluoview FV300, Olympus, Milan, Italy) after the immunostaining of specific neuronal markers, such as β-tubulin III, a protein associated with the cytoskeleton that is present in the soma and in all neuronal extensions; growth-associated protein-43 (GAP 43), a specific component of axonal extensions; and synaptophysin, a synaptic vesicles marker. Neurons were fixed in 4% (wt/v) paraformaldehyde for 15 min, permeabilized with 0.3% Triton X-100 in PBS for 10 min and then blocked with 1% (*v*/*v*) BSA for 30 min at room temperature. Then, samples were incubated overnight at 4 °C with the following primary antibodies: rabbit anti-βTub III (1:500, Covance, Princeton, NJ, USA), mouse anti-synaptophysin (1:400, Chemicon, Millipore, Burlington, MA, USA) and goat anti-GAP-43 (1:200, Santa Cruz Biotechnology, Dallas, TX, USA). Secondary antibodies, Cy2^TM^-conjugated Affini Pure donkey anti-rabbit IgG, Cy3^TM^-conjugated Affini Pure donkey anti-mouseIgG and a Cy5^TM^-conjugated Affini Pure donkey anti-goat IgG (1:500, Jackson ImmunoResearch Europe Ltd., Cambridge, UK), were added for 1 h at RT. Finally, cells were counterstained with 200 ng/mL DAPI (Molecular Probes) for nuclear localization. Samples were rinsed, mounted and observed at LSCM.

#### 2.3.3. Metabolic Function

The metabolic activity of neuronal cells was evaluated by investigating the glucose consumption in the supernatants of neuronal cultures collected during the culture time within the membrane system. Glucose concentration and consumption were detected by using Accu-Chek Active glucose assay (Roche Diabetes Care, Monza, Italy).

### 2.4. Cell Treatments: Oxidative Stress, β-Amyloid Toxicity and Trans-Crocetin Administration

To realize an in vitro model of oxidative stress, differentiated neuronal cells, after 7 days of culture in PLGA membrane system, were exposed to hydrogen peroxide (H_2_O_2_; Sigma-Aldrich) diluted in culture medium at a concentration of 150 μM, for 24 h at 37 °C.

The antioxidant molecule tert-Butylhydroquinone (tBHQ; Sigma-Aldrich) (40 μM) was used as reference control.

The toxic model of Alzheimer’s disease was created within the membrane system by treating the differentiated neuronal cells with a culture medium containing 5 μM of β-amyloid_1–42_ (Aβ) peptide-HFIP (Anaspec, Seraing, Belgium), for 24 h at 37 °C, in accordance with previous studies [8].

To investigate the neuroprotective effects of trans-crocetin (Extrasynthese, Genay, France) against H_2_O_2_/Aβ-induced toxicity, cells were treated with a mixture of H_2_O_2_ (150 μM) or Aβ (5 µM) and trans-crocetin at different concentrations (10, 20 and 50 μM) for 24h at 37 °C. Trans-crocetin was dissolved in EtOH to create a 2 mM stock solution and added to the culture medium at the indicated concentrations.

We used the nomenclature “Control” to indicate untreated cells without toxic insult (H_2_O_2_ or Aβ) and trans-crocetin treatment. After 24 h under the different treatments, cells were sacrificed for assessment of neuronal response and the neuroprotective effect of trans-crocetin. All experiments were conducted in triplicate with the average of 5 samples for treatment (Control, H_2_O_2_/Aβ insult and co-treatments with trans-crocetin).

### 2.5. Evaluation of Trans-Crocetin Anti-Oxidant Effect

Trans-crocetin’s capacity to inhibit H_2_O_2_-induced oxidative stress was investigated by assessing neuronal viability and intracellular reactive oxygen species (ROS) production.

#### 2.5.1. Cell Viability

The viability of neuronal cells was measured using the colorimetric assay based on the reduction of a yellow tetrazolium salt (3-(4,5-dimethylthiazol-2-yl)-2,5-diphenyltetrazolium bromide or MTT) to purple formazan crystals by metabolically active cells.

Neuronal cells, after 24 h of treatment with the different concentrations of trans-crocetin and t-BHQ in the presence of H_2_O_2_, were incubated with a solution of MTT (5mg/mL) for 2 h at 37 °C. The reaction was blocked by adding a lysis buffer (10% of SDS, 0.6% acetic acid in dimethyl sulfoxideDMSO- pH 4.7) that allows for lysing of the living cells, thus favoring the release of formazan. Finally, the absorbance of formazan produced by cells was spectrophotometrically measured at 570 nm. The degree of cell viability in terms of mitochondrial activity was expressed as a percentage of the control without any treatment.

#### 2.5.2. ROS Production

The generation of reactive oxygen species (ROS) was detected using the oxidant-sensing non-fluorescent probe 2′,7′-dichlorodihydrofluorescein diacetate (H_2_DCF-DA). The molecule is converted into the polar derivative nonfluorescent DCFH by cellular esterases, and then switched to highly fluorescent DCF when oxidized by intracellular ROS. Therefore, DCF’s fluorescence intensity is an index of intracellular ROS levels. In our experiments, after treatment with the different concentrations of trans-crocetin and t-BHQ in the presence of H_2_O_2_, the cultured medium was removed, and 50 μM of H_2_DCFDA was added to each sample and incubated for 30 min at 37 °C. Cells were then washed with Hank’s Balanced Salt Solution, and finally the fluorescence’s intensity of DCF was measured with LSCM by using an argon laser. Quantitative analysis was performed on LSCM images of cells using Fluoview 5.0 software.

### 2.6. Detection of Trans-Crocetin Anti-β-Amyloid-Induced Toxicity

#### Apoptosis Detection

For mitochondrial membrane potential measurement, the mitochondrial membrane potential (MMP) was measured with the fluorescent cationic probe 5,5′,6,6′-tetrachloro-1,1′,3,3′-tetraethylimidacarbocyanine iodide (JC-1) (Sigma-Aldrich, St. Louis, MO, USA). In healthy cells with high MMP, JC-1 dye aggregates and stains the mitochondria with red fluorescence, whereas in cells with low MMP, the dye remains in the cytoplasm in a green fluorescent monomeric form. Therefore, mitochondrial depolarization is indicated by a reduction in the red to green JC-1 fluorescence intensity ratio. Cell cultures were incubated with 50 μg/mL JC-1 for 20 min at 37 °C. Next, the cells were washed, and red and green fluorescence intensities were measured by LSCM using a He/Ne green laser and Ar laser, respectively. Then, a quantitative analysis was performed using Fluoview 5.0 software, and data were expressed as ratios of red (aggregate form) to green (monomeric form) fluorescence intensity.

In terms of Caspase 3 and Jun protein kinase (JNK) expression, the expression of two specific apoptotic markers, Caspase-3 and p-JNK, were analysed with LSCM after immunostaining using anti-Caspase-3 antibody (1:250, Becton, Dickinson and Company, Franklin Lakes, NJ, USA) and anti-p-JNK antibody (1:250, Santa Cruz Biotechnology). Quantitative analysis was performed by calculating the percentage of cells positive for the distinct apoptotic marker (Caspase-3 positive or p-JNK-positive cells) over total nuclei (DAPI-stained nuclei)

### 2.7. Statistical Analysis

Statistical significance of the experimental results was calculated using an ANOVA test followed by a Bonferroni *t*-test (*p* < 0.05).

## 3. Results and Discussion

### 3.1. Characterization of PLGA Membrane Properties

The most challenging aspect of providing a nerve tissue model is the reproduction of the exact microcellular environment and cell–cell contacts. With the aim to address this critical issue, tissue engineering and regenerative medicine approaches provide alternative paths. These methods mainly include the use of biomimetic interfaces able to promote cell migration and tissue repair. In this context, neuronal tissue engineering offers a wide variety of alternative approaches for reliable a platform in which neurons’ viability and specific functions are retained. Neuronal tissue engineering therefore provides useful models in which neurological disorders can be mimicked. The use of these systems for disease modelling represents a valid substitute to the animal model for testing new therapeutic molecules. Thus, they can serve as a wide screening plan in order to create an initial overview of neuronal behaviour in response to a specific therapeutic treatment. Here, we present a PLGA multiplex membrane platform, which features several fundamental prerequisites for an efficient investigational platform. It consists of a continuous array of PLGA membrane chambers, where each membrane spot allows for dedicated investigation inherent to the case of study. The possibility of performing different and simultaneous experimentation within the same device speeds up the final outcomes with overall improvement in terms of cost and insights gained. The realization of a membrane in vitro platform implies the initial selection of polymeric membranes that better drive cellular adhesion and differentiation according to the main features of the engineered tissue.

When cells are seeded over a bioactive membrane, they immediately need to re-organize themselves, and the interfacial interaction between the membrane’s surface and neuronal cells is therefore essential to ensure their survival and afterwards their specialized functions. Cell–membrane attachment is strictly related to cell culture media protein attachment, the presence of which is responsible of the subsequent cell membrane binding. Protein adsorption implicates molecular-scale interactions with the membrane surface, and therefore surface properties play a pivotal role in this process. Only after an appropriate protein adsorption at the membrane interface will cell adhesion and cell spreading take place. To assess PLGA membrane suitability as the appropriate matrix within the multiplex membrane platform, its structural, physico-chemical and mechanical properties were characterized (Table 1). Morphological observation through SEM analysis pointed out a homogeneous membrane surface (data not shown), while the flow porometer investigation revealed a mean pore size of 16 ± 2 nm. Membrane wettability features have been explored through water dynamic contact angle measurements, which evidenced a moderate hydrophilic character with an advancing contact angle of 89 ± 4° and a receding one of 55 ± 2°; the hysteresis value is 34°. Interface with a moderate wettability enables cells adhesion, proliferation and spreading as a consequence of the promoted ability to bind a range of biomolecules [20]. Indeed, a highly wettable surface would not favour biomolecule interaction, thus hindering cell adhesion. An intense hydrophobic profile would instead determine a local excess of protein deposition and possible conformational changes. Such atypical protein deposition could compromise proper interaction with the plasma membrane recognition binding sites, responsible for cell adhesion and spreading over the membrane surface. The moderate wettability features that characterize the PLGA membrane ensure an optimal cell–membrane interaction for the development of intense neuronal arborization. The neuronal attachment process is influenced by mechanical and biophysical interactions determined by the membrane stiffness. Indeed, neuronal cells can sense the membrane stiffness, which acts as mechanical stimulus that evokes a mechanotransduction; this includes several pathways, like the formation of focal adhesions, which are fundamental for correct cell–cell interaction [21]. Membrane mechanical properties characterization was therefore performed in order to disclose another crucial parameter that affects cell behaviour. The membrane tensile stiffness, represented by the Young modulus [E], is 293 ± 76 N/mm^2^. Besides stiffness, ultimate tensile strength (UTS) and elongation at break [ε] also influence the overall biomechanical characteristics of the PLGA membrane, which have been defined and reported in Table 1. In a previous work, aimed at disclosing the effect of membrane mechanical properties for the reconstruction of a neuronal tissue analogue [15], we showed that UTS and elongation at break are two key parameters able to influence growth cone formation and axonal and dendritic complex arborization. The mechanical features of brain tissue are quite peculiar, being anisotropic and extremely soft (e.g., <1 kPa) [22]. Although the evident mechanical mismatch between the PLGA membrane and the native brain tissue PLGA, membrane mechanical features fit within the range of other material [1] and substrate used in neuronal tissue engineering (e.g., few hundred Pa for hydrogels, 1–2 GPa for tissue culture polystyrene).

PLGA mechanical characterization corroborates its suitability as an interfacial biomembrane in contact with neuronal cells within the multiplex membrane platform.

### 3.2. PLGA Multiplex Membrane Platform Enables Neuronal Growth and Differantiation

**Figure 2:**
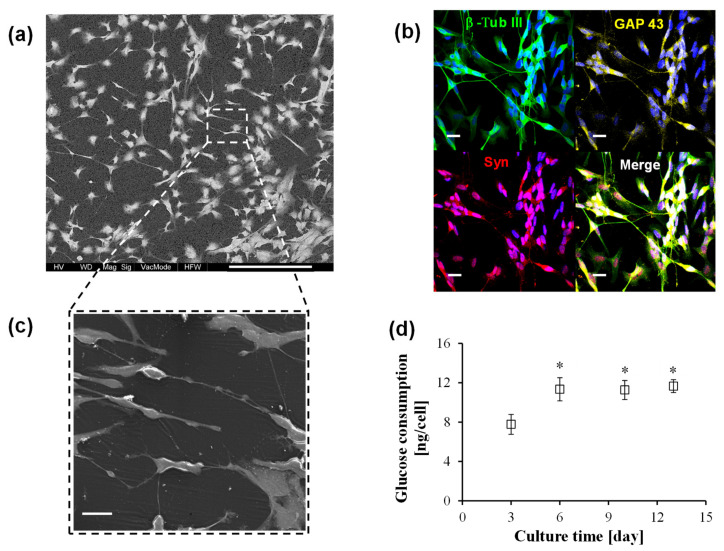
Neuronal differentiation in PLGA multiplex membrane platform: (**a**) and (**c**) are SEM micrographs at different magnifications (scale bar = 200 µm and 20 µm, respectively), and (**b**) is a confocal laser scanning microscope image of neuronal cells after 14 days of culture in the membrane system (scale bar = 20 µm); cells were stained for neuronal markers βIII-tubulin (green), synaptophysin (red), Growth Associated Protein 43 (GAP-43, yellow) and cell nuclei marker (DAPI, blue); (**d**) glucose consumption of neurons were cultured within the PLGA membranes device up to 14 days; the values, expressed as average ± SD, are the means of three experiments, and statistical analysis was performed according to an ANOVA followed by a Bonferroni *t*-test (*p* < 0.05): * vs. day 3.

The structural, mechanical and physico-chemical properties of the PLGA membrane give an encouraging hint of its great potential in neuronal tissue engineering. The use of an in vitro platform for the study of health and diseased neurons is valid only when nervous tissue reconstruction recapitulates both structural and functional features. To meet this demand, the bioactive membrane within the investigational tool has to offer a cell-friendly environment; only after the accomplishment of this issue it can serve for the screening of new therapeutic molecules. Before using the proposed platform for the evaluation of trans-crocetin treatment over insulted neurons, we verified the ability of the membrane system to guide and maintain the arrangement of neuronal highly branched projections, which are good indicators of a proper synaptic organization. SEM images of neurons cultured on the PLGA membrane for 14 days (Figure 2a–c) show a good interaction with the membrane’s surface. They tightly adhered over the new PLGA matrices, covering the whole membrane area. The appropriate structural organization is evidenced by intense neurite extension with multiple directions, giving rise to a complex network. Extending axon and dendritic arborization, visible at higher magnification in Figure 2c, appear very close to each other and thus, possibly, synaptic contacts can be formed. The structural and functional integrity of neuronal cells was further investigated by using confocal microscopy analysis, allowing the visualization of specific neuronal markers (Figure 2b). Dynamic and functional microtubules are essential for correct neuronal process outgrowth. The cytoskeleton integrity of neurons over the PLGA membrane is corroborated by the wide β-Tub III distribution visualized in green. This specific protein is visible both in the cell body and in the neural extensions, ensuring the maintenance of the specialized morphology. GAP 43 is a protein involved in the development of neuronal growth cones and axonal regeneration. Its consistent expression in neurons kept in culture in the PLGA membrane platform indicates that this system does not only promote neuronal survival but also preserves their plasticity. As marker of the retained specialized functions, we selected synaptophysin, which is a protein highly concentrated within presynaptic vesicles. Synaptophysin localization confirms that neurons cultured over PLGA membranes reached a proper degree of maturation. Glucose, which is the main source of energy for neurons, was continuously consumed by cells as an indication of their active metabolism over the culture time (Figure 2d). The developing neural network has high energy demand, as reflected by the rapid increase in terms of the sugar consumption up to the sixth day of culture. Afterwards, the trend of glucose uptake is quite high and constant to supply the huge metabolic demand necessary to sustain neuronal survival, synaptic transmission and information processing. These findings demonstrate that the PLGA membrane system enables neuronal growth and differentiation, allowing for the formation of a functional neuronal-like tissue analogue.

### 3.3. PLGA Multiplex Membrane System Allows Multiple Simultaneous Investigation

The PLGA multiplex membrane system was employed as a therapeutic screening platform to assess the potential neuroprotective effect of the carotenoid trans-crocetin. Specifically, to validate the system as a reliable device that allows for multiple investigations, two key neuro-pathological pathways of Alzheimer’s disease development, namely oxidative stress and β-amyloid toxicity, were modelled simultaneously within the PLGA membrane device. Then, the antioxidant and anti-amyloidogenic potentials of trans-crocetin were assessed at the same time.

#### 3.3.1. Anti-Oxidative Effect of Trans-Crocetin by Means of Membrane Platform

Neurons are particularly susceptible to oxidative stress damage, which is primarily involved in the pathogenic onset and progression of neurodegeneration including Alzheimer’s disease (AD). Oxidative stress is caused by free radicals that contain one or more unpaired electrons. Among the radicals, the main molecules implicated in oxidative damage are oxygen reactive species (ROS) such as superoxide anion and hydroxyl radical and hydrogen peroxide (H_2_O_2_). An increase in these molecules within the body causes damage to vital biomolecules such as membrane phospholipids, with loss of selectivity, cellular protection, proteins, and enzymes; impairment of metabolic reactions and nucleic acids; accumulation of mutations; and alterations of gene expression.

In keeping with previous studies, differentiated neurons within the PLGA multiplex membrane system were treated with H_2_O_2_ as an oxidizing agent, to model oxidative stress in order to investigate the antioxidant role of trans-crocetin.

Cells in normal condition have a complex system of intracellular antioxidant defenses that preserve a physiological redox balance. When an imbalance between the production of ROS and the effectiveness of the antioxidant endogenous system occurs, an oxidative stress condition is generated, which can irreversibly damage cellular functionality. Therefore, antioxidants of an exogenous origin, such as bioactive molecules present in foods, mainly of plant origin, appear to be protective agents against the different diseases caused by oxidative damage, such as neurodegenerative diseases including AD. Recent works have suggested the antioxidant effect of carotenoids present in saffron (*Crocus sativus L.*) [23] as a possible solution. In particular, the carotenoids trans-crocetin and crocin are the major bioactive constituents of saffron with neuroprotective potential [24]. It has been demonstrated that trans-crocetin is responsible for the main therapeutic properties of saffron [25]. However, the molecular mechanism by which they perform a neuroprotective effect is not fully defined. Whilst a large body of research has been conducted on the protective action of crocin in the treatment of AD [6,26], the role of trans-crocetin in such a disease is not yet fully understood and needs to be elucidated. So, in an attempt to clarify the neuroprotective effect of trans-crocetin, we carried out experiments that aimed to analyze whether this carotenoid protects neurons against H_2_O_2_-induced neuronal damage, evidencing a significant role in blocking oxidative stress.

**Figure 3:**
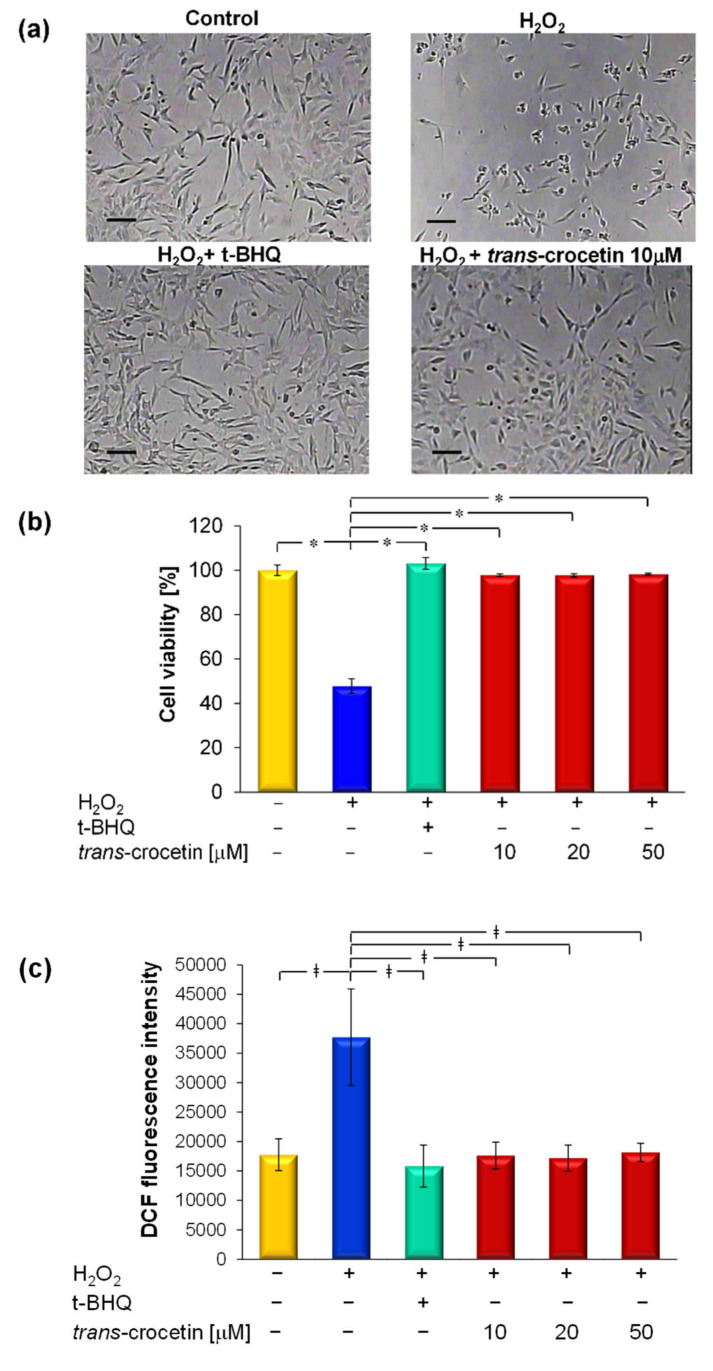
Trans-crocetin neuroprotective effect against H_2_O_2_-induced oxidative stress: (**a**) representative images of neurons in the PLGA multiplex membrane system without any treatment (control), after 24 h of hydrogen peroxide insult (H_2_O_2_), co-treatment with ter-butyldroquinone (t-BHQ) (H_2_O_2_ + t-BHQ) and trans-crocetin (10 µM) (H_2_O_2_ + trans-crocetin); (**b**) cell viability and (**c**) intracellular oxygen reactive species(ROS) levels, calculated by quantitative analysis of Dichlorofluorescein (DCF) fluorescence intensity, in neuronal cells under different treatments. The antioxidant tBHQ was used as a positive control. The values expressed as average ± SD are the means of three experiments, and statistically significant data were evaluated according to an ANOVA followed by a Bonferroni *t*-test (*p* < 0.05). * vs. H_2_O_2_ insult, ǂ vs. other treatments.

Morphological observation (Figure 3a) revealed how H_2_O_2_-damaged neurons obviously underwent to cell death, and the consequent cell loss as evidenced by the wide empty areas over the PLGA membrane surface. H_2_O_2_-insulted neurons have also been cotreated with trans-crocetin in a concentration of 10 µM. Carotenoid administration protected cells from structural disruption, allowing the preservation of neural integrity and thus neutralizing H_2_O_2_ action. To confirm trans-crocetin antioxidant activity, we compared its effects with the ones evoked by ter-butyldroquinone (t-BHQ), whose antioxidant activity is well known in the literature [27]. t-BHQ has therefore been used as a reference compound. The comparison resulted in the acquisition of very similar images, which reflected the same neuroprotective potential for both molecules. A more quantitative estimation of the neuroprotective effect of trans-crocetin against H_2_O_2_ damage is provided by a viability test of the increasing concentration of trans-crocetin (Figure 3b). In agreement with previous reports [17,18], the investigation evidenced that this natural molecule is able to counteract H_2_O_2_ neurotoxicity, preserving cell viability. As expected, oxidative stress due to treatment with H_2_O_2_ caused a significant decrease of cell viability of 48% ± 3%. In contrast, the co-treatment with trans-crocetin induced a significant increase of cell viability to a value that is fully comparable with the one obtained in the control (100% ± 3%) and by co-treating cells with reference molecule t-BHQ (103% ± 3%). Moreover, trans-crocetin already offered neuroprotective effects at a low concentration of 10 µM (98% ± 1%), preventing neuronal cell death caused by H_2_O_2_ insult.

The antioxidant activity of trans-crocetin, related to the toxicity induced by H_2_O_2_, was further investigated by LSCM analysis, exploiting the fluorescence of oxidized DCF, which is related to the intracellular ROS generation. Quantitative analysis (Figure 3c) showed that ROS levels increased significantly in the presence of H_2_O_2_, thereby indicating the induction of oxidative stress.

Trans-crocetin treatment at different concentrations induced a significant decrease of DCF fluorescence intensity that resulted in a decrease of the intracellular ROS production and accumulation; this kind of analysis again confirmed that the lowest concentration of molecules protects from oxidative stress induced by H_2_O_2_. These findings are consistent with previous work by Papandreou et al., who reported that trans-crocin is effective in inhibiting intracellular ROS accumulation caused by H_2_O_2_ toxicity in neuroblastoma cells [28].

For the first time in the literature, our work provides evidence of trans-crocetin’s role in scavenging H_2_O_2_-induced ROS production in a similar trend observed for the reference antioxidant molecule t-BHQ, demonstrating both neuroprotective and antioxidant actions mediated by trans-crocetin.

#### 3.3.2. Anti-Amyloidogenic Effect of Trans-Crocetin by Means of Membrane Platform

In addition to the oxidative stress model, the platform was simultaneously used to investigate trans-crocetin’s protection against Aβ-induced neuronal impairment. Amyloid β peptide (Aβ) is the primary constituent of the amyloid plaque found in the brain of patients with AD and represents a validated therapeutic target for AD intervention. Therefore, differentiated neurons within the PLGA multiplex membrane platform were treated with Aβ (1–42) (5 µM) for 24 h to create an in vitro model of the neurotoxicity associated with AD.

Since the aggregation of Aβ peptide induces neuronal damage that leads to the cell apoptosis implicated in the onset of AD, we investigated the ability of trans-crocetin in preventing Aβ-induced apoptosis to disclose its the neuroprotective mechanism of action.

**Figure 4:**
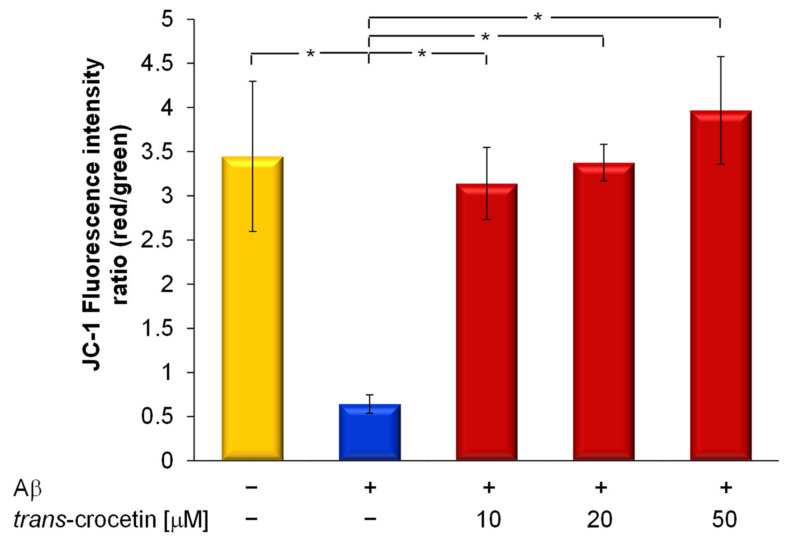
Trans-crocetin inhibited Aβ-induced depolarization. Mitochondrial membrane potential was detected by the ratio between JC-1 red and green fluorescence intensity. The values, expressed as average ± SD, are the means of three experiments, and statistically significant data were evaluated according to an ANOVA followed by a Bonferroni *t*-test (*p* < 0.05). * vs. Aβ-insult.

Considering the early stages of apoptosis are characterized by the loss of mitochondrial membrane potential (MMP), we investigated the effect of trans-crocetin on the Aβ-induced decrease of MMP using a potential-sensing fluorescence probe (JC-1) in LSCM. The fluorescent intensity ratio value (red/green) of JC-1 in neuronal cells was significantly decreased (*p* < 0.05) with the addition of 5 µM Aβ, demonstrating the decrease of MMP in neuronal cells (Figure 4) and thereby inducing impairment in mitochondrial function. Trans-crocetin suppressed Aβ-induced mitochondrial dysfunction, as proved by the significant increasing of the fluorescent intensity ratio, which reaches values close to those measured in the control cells without any treatment.

In a previous study, trans-crocetin did not prevent the loss of mitochondrial membrane potential in a model of retinal damage [18]; in contrast, more recently, Kong et al., indicated its protective action against Aβ-induced mitochondrial damage [29]. Our study is consistent with the latter work and thus provides further important evidence of neuroprotective effect caused by the carotenoid.

**Figure 5:**
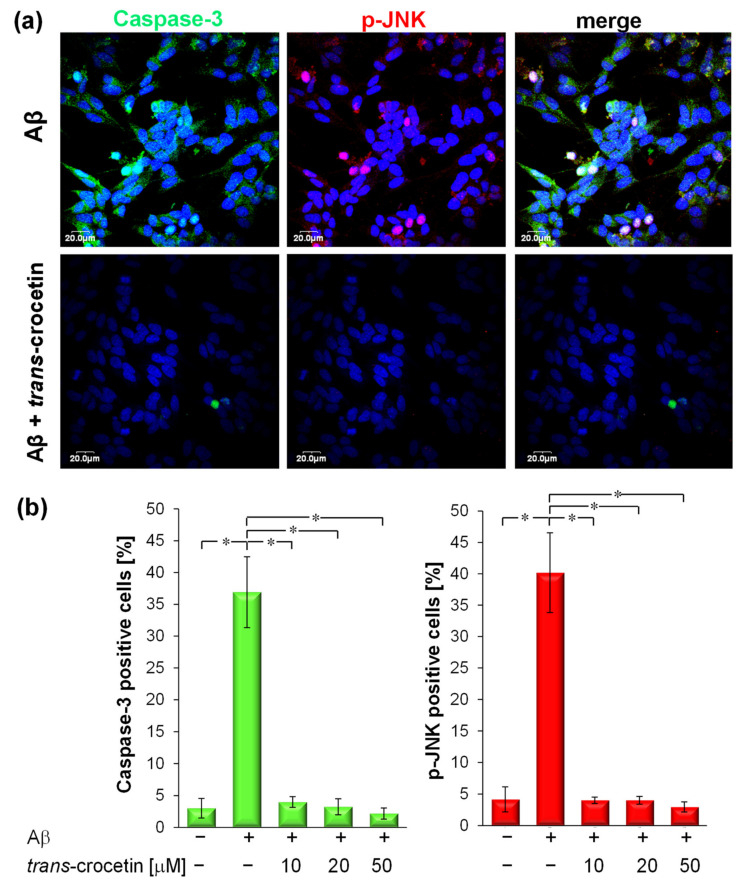
Trans-crocetin defended neurons against Aβ-induced apoptosis: (**a**) Confocal laser representative micrographs of neurons in the PLGA multiplex membrane platform after Aβ treatment and after co-treatment with Aβ and trans-crocetin 10 μM. Cells were stained for apoptotic markers active Caspase-3 (green) and p-JNK (red), and nuclei (blue). (**b**) Quantitative analysis of apoptotic markers. The values, expressed as average ± SD, are the means of five experiments, and statistically significant data were evaluated according to an ANOVA followed a Bonferroni *t*-test (*p* <0.05): * vs. other treatments.

The apoptotic process is regulated by many intracellular enzymes, including caspase-3 and phosphorylated Jun protein kinase (p-JNK), whose activation and cleavage may be implicated in the early events involved in Aβ toxicity. Consequently, we have investigated the ability of trans-crocetin to modulate the activation of these apoptotic markers, which reflects the hierarchical position of these proteins in the sequence of events leading to apoptosis. In particular, p-JNK represents the upstream sensor protein, and Caspase-3 represents the effector, a downstream protein, of the apoptotic pathway. Apoptotic cells were thus detected by immunofluorescence analysis in LSCM assays (Figure 5a), evidencing a perinuclear and nuclear localization of the two apoptotic markers [30,31]. The percentage of positive cells was calculated by the ratio of apoptotic nuclei over total nuclei (Figure 5b).

Aβ insult produced a significant increase in levels of cleaved Caspase-3 (37 ± 5%) and phosphorylated JNK (40 ± 6%), which are visible as green and red regions, respectively, in Figure 5a. As depicted in such images, the co-administration of trans-crocetin modulated both signaling proteins, inhibiting the expression and thus the activation of both apoptotic markers. When trans-crocetin (10–50 µM) was present, the Aβ-induced increase of cleaved Caspase-3 and phosphorylated JNK was completely prevented, similarly to the control experiment, meaning that there was a protective effect of trans-crocetin against Aβ-induced cell apoptosis (Figure 5b).

A previous study by Yamauchi et al. indicated that trans-crocetin inhibited the activation of Caspase-3 induced by H_2_O_2_ in retinal ganglia cells [18]. The present study is, to our knowledge, the first report to provide evidence of trans-crocetin’s ability to inhibit the enzymatic activity of Caspase-3 and p-JNK in neurons after treatment with Aβ peptide.

Our findings highlight that treatment with trans-crocetin can reverse a series of neurotoxic effects caused by H_2_O_2_ and Aβ treatments, supporting its preventive and possible therapeutic potential against AD.

The capacity of trans-crocetin to counteract both oxidative stress and Aβ-induced toxicity could be attributed to its chemical structure. The scavenging activity is due to the presence of a hydroxyl moiety in the carboxylic group, which reacts with free radicals. Also, the presence of sugar groups, attached to the terminal -COOH of trans-crocetin’s backbone allows extensive distribution and penetration of trans-crocetin through lipid bilayers of cell membranes [32]. In addition, the negatively charged carboxyl groups and the hydrophobic hydrocarbon chain of trans-crocetin can interact with positively charged amino acids and the hydrophobic sequence at the C-terminal domain in Aβ, respectively [23], thereby inhibiting Aβ aggregation.

Our findings, together with the consideration of trans-crocetin’s ability to penetrate the blood−brain barrier [33], highlight the potential value of such a natural agent to counteract AD progression. However, in vivo trial studies need to be performed to further demonstrate its effectiveness as therapeutic for AD.

## 4. Conclusions

In this study, we realized a multiplex membrane device, consisting of a continuous array of PLGA membrane chambers, as an innovative platform for performing different and simultaneous investigation.

Morphological, structural, physico-chemical and mechanical properties of PLGA membranes offered proper support, guiding cell adhesion, growth and neuronal differentiation. Our tool therefore represents a good compromise between a well-mimicked brain tissue analogue combined with an easily accessible and implemented membrane system.

Overall, our study validates the PLGA multiplex membrane system as a reliable device that allows us to model different neuropathological events for multiple simultaneous investigations. It enables us to demonstrate the role of trans-crocetin double protective action in defending neurons against both oxidative stress and Aβ-induced toxicity, which play important role in the pathogenesis of AD. Trans-crocetin counteracted the deleterious effects on neuronal viability mediated by H_2_O_2_ treatments and scavenged H_2_O_2_-induced ROS production. At the same time, it protected from Aβ-induced neurotoxicity by preventing mitochondrial dysfunction and suppressing cell apoptosis caused by Aβ treatment.

Our findings highlight the potential value of such a natural multifunctional agent in combating AD. Future in vivo trial studies need to be performed to further demonstrate its effectiveness as therapeutic for AD.

## Figures and Tables

**Table 1:** Poly(lactic-co-glycolic acid) (PLGA) membrane properties. Advancing (θadv) and receding (θrec) water contact angle (WCA); Young’s modulus (E); elongation at break (ε) and ultimate tensile strength (UTS).

PLGA Membrane	
Thickness [μm]	14 ± 2
Mean Pore Diameter [nm]	16 ± 2
WCA [°]	θadv 89 ± 4 θrec 55 ± 2
E [N/mm^2^]	293± 76
ε [%]	397 ± 40
UTS [N/mm^2^]	15 ± 31

## Data Availability

Data is contained within the article.
